# Combination of intrahepatic TARE and extrahepatic TACE to treat HCC patients with extrahepatic artery supply: A case series

**DOI:** 10.1016/j.redii.2024.100042

**Published:** 2024-03-10

**Authors:** Lorenzo Carlo Pescatori, Athena Galletto Pregliasco, Haytham Derbel, Laetitia Saccenti, Mario Ghosn, Maxime Blain, Julia Chalayea, Alain Luciani, Sebastien Mulé, Giuliana Amaddeo, Hicham Kobeiter, Vania Tacher

**Affiliations:** a Department of Interventional Radiology, hôpital Avicenne (AP-HP), Bobigny, France; b Department of Radiology, hôpital Henri-Mondor (AP-HP), Créteil, France; c Inserm IMRB U955, équipe 18, université Paris-Est Créteil, Créteil, France; d Department of Nuclear Medicine, hôpital Henri-Mondor (AP-HP), Créteil, France; e Department of Hepatology, hôpital Henri-Mondor (AP-HP), Créteil, France; f Inserm IMRB U955, équipe 8, université Paris-Est Créteil, Créteil, France

**Keywords:** TACE, TARE, Extrahepatic artery, HCC

## Abstract

**Purpose:**

The aim of this study was to report the safety and tumor response rate of combined transarterial radioembolization (TARE) through the intrahepatic arteries and transarterial chemoembolization (TACE) through the extrahepatic feeding arteries (EHFA) in patients with hepatocellular carcinoma (HCC).

**Methods:**

Patients with HCC, who had both intrahepatic and extrahepatic arterial supply visible on preinterventional multiphase CT and were treated between 2016 and 2021 with a combination of TACE and TARE on the same nodule, were retrospectively included. Epidemiological, clinical, biological, and radiological characteristics were recorded. Safety and tumor response were assessed at 6 months.

**Results:**

Nine patients (8 men, median age 62 years [IQR: 54–72 years]) were included. Seven patients had previous treatments on the target nodule (TARE: 5; TACE: 2). The median longest axis (LA) of the lesion was 70 mm (IQR: 60–79 mm). Three patients had portal vein invasion (VP3). The EHFA originated from the right diaphragmatic artery (*n* = 6), the right adrenal artery (*n* = 2), and the left gastric artery (*n* = 1). The LA of the tumor portion treated with TACE was 47 mm (range: 35–64 mm). The ratio between the LA of the entire lesion and the LA treated with TACE was 1.44 (range: 1.27–1.7). One major complication occurred: acute on chronic liver failure. Median follow-up was 23 months (range: 16–29 months). Seven patients underwent further treatment: on the same lesion (*n* = 2), on newly appeared nodules (*n* = 2), and systemic treatment (*n* = 3). At 6-month follow-up, seven patients showed a local objective response. Time-to-progression was 13 (3.5–19) months.

**Conclusion:**

The combination of TARE and extrahepatic TACE for HCC with both intrahepatic and extrahepatic arterial supplies seems feasible and safe. Further studies are needed to validate the effectiveness of these preliminary results.

## 1 Introduction

Transarterial radioembolization (TARE) was included in the 2022 Barcelona Clinic Liver Cancer (BCLC) guidelines as a therapeutic option for patients classified as BCLC stages 0 and A. This recommendation specifically applies to cases where ablation or resection is not feasible and only for lesions smaller than 8 cm in their longest axis. Furthermore, according to recommendations from various national and international societies, such as the European Society for Medical Oncology (ESMO) [1] or the National Institute for Health and Care Excellence (NICE) [2], TARE also plays a role in the palliative treatment of patients classified as stage B or C according to the BCLC classification. This application of TARE is particularly relevant for patients without extrahepatic lesions who have contraindications to systemic therapy or transarterial chemoembolization (TACE). The technique itself does not differ from TACE, but, as the local tumor control depends on a high dose of radiation delivered to the tumor, selective catheterization of the arteries feeding the tumor is needed to minimize the risk of non-target damage to the surrounding liver parenchyma [3], [4], [5], [6]. Moreover, as TARE can be proposed as a salvage therapy for lesions over 5 cm in diameter, most patients have already had several previous treatments: i.e., surgery, systemic treatment, ablations, or TACE [7], [8], [9], [10], [11]. Thus, lesions may have arterial abnormalities induced by tumor progression or previous embolizations, leading to the recruitment of extrahepatic feeding arteries (EHFA), especially in the case of peripherally located tumors [12], [13], [14]. However, TARE cannot be performed through an EHFA as the risk of delivering radiation to non-target territories is elevated and burdened by major complications [13]. Thus, embolization of EHFAs has been proposed to redistribute arterial flow to the liver lesion [12], [13], [14], facilitating effective redistribution of the radiation dose during TARE performed solely through the hepatic arteries. Essentially, two techniques have been proposed: coiling or embolization with particles of the EHFA [12,14,15].

The first option was described as being ineffective due to the opening of shunts around the EHFA [14,15]. Conversely, the second option seems reliable in redistributing the flow to the intrahepatic branches, but it risks occluding the vascularization to a part of the lesion [12] which consequently will not be treated during TARE and thus not receive the appropriate radiation dose.

Herein, we report a series of patients affected by HCC with EHFAs who underwent TARE through the intrahepatic vessels and TACE through the EHFA in order to evaluate the safety, feasibility, and tumor response rate of the combined treatment.

## 2 Materials and methods

This is an observational, single-institution, retrospective study concerning patients with HCC treated with concomitant TARE and TACE through intra- and extrahepatic approaches between March 2016 and March 2021.

Informed consent was obtained from every patient, and medical ethics approval was waived due to the retrospective nature of the study.

HCC was diagnosed either by biopsy or based on imaging characteristics, following the guidelines of the European Society for the Study of the Liver (EASL) [16].

The indication for TARE was given after a multidisciplinary meeting involving hepatologists, oncologists, surgeons, and radiologists. The decision was based on the non-resectability of the lesion due to its dimensions, position, or clinical risks associated with surgical treatment.

A contrast-enhanced CT scan (CECT) is routinely performed at our institution for patients selected for endovascular treatment of HCC. EHFAs were identified, paying particular attention to tumors in contact with the diaphragm or near the right or inferior border of the liver, due to the possibility of vascularization through the phrenic, intercostal, adrenal, or lumbar arteries as previously described in the literature [12] If an EHFA supplying the lesion was not found at the preliminary CECT, its presence could be suspected on the yttrium 90 (^90^Y) PET/CT obtained after TARE, in cases where a subcapsular circumscribed area lacked microsphere distribution. In these cases, TACE was performed in a subsequent session. Patients were included if they had TARE and TACE on different portions of the same lesion, either in the same session or within a 3-month interval.

Demographic and clinical characteristics were obtained for the selected population, including age, gender, cause of liver cirrhosis (if any), tumor morphology, and previous liver treatments.

BCLC, Child-Pugh, and Eastern Cooperative Oncology Group (ECOG) scores [17], as well as laboratory parameters (i.e., alpha-fetoprotein [AFP], serum albumin, prothrombin time [PT], aspartate transaminase [AST], alanine transaminase [ALT], and total bilirubin) were collected before treatment and during follow-up. BCLC was recalculated based on the version updated in 2022 [18].

### 2.1 TARE

**Fig. 1:**
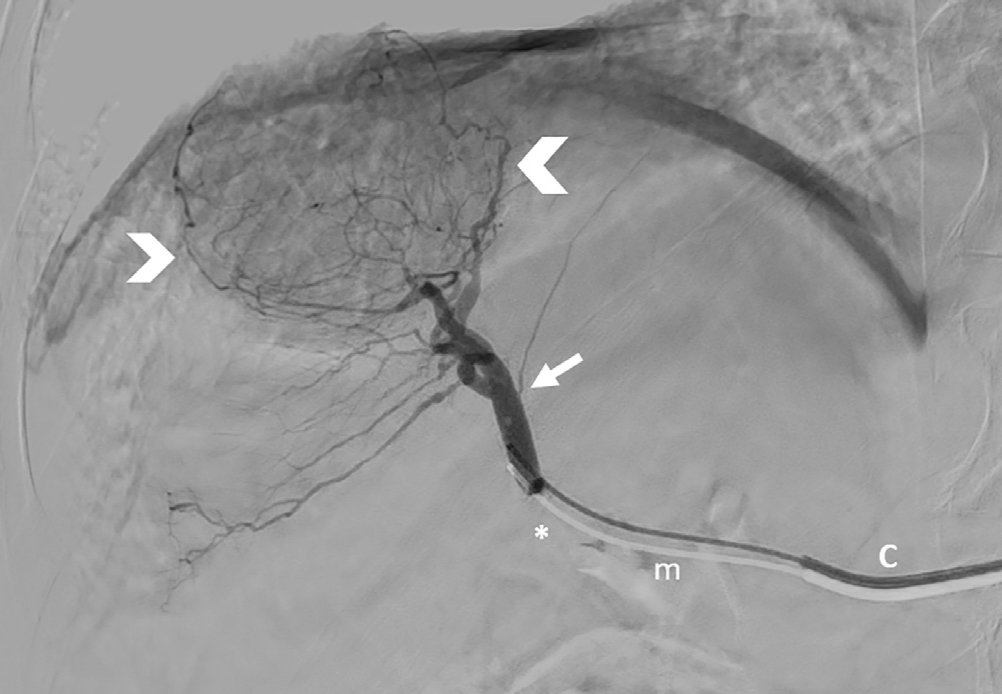
Digital subtraction angiography (DSA) at the bifurcation (asterisk) of the posterior branch of the right hepatic artery (arrow) showing the vascularisation of the main part of the liver lesion (arrowheads). C: 5-french catheter, m: 2.4-french microcatheter.

All treatments were performed by a senior radiologist with at least 2 years of experience in endovascular procedures. TARE was performed in two sessions, as per the conventional model, with a workup phase and a treatment phase. The workup aimed to find the exact point of injection to cover the whole lesion in the treatment field while minimizing non-tumoral parenchyma [Fig. 1]. The correct positioning of the microcatheter was always confirmed with a dual-phase cone-beam computed tomography (DP-CBCT), and followed by a slow injection of technetium (^99^mTc) albumin aggregated ([^99^mTc]-MAA). The patient then underwent a SPECT/CT within 1 h after the workup to check for any extrahepatic diffusion of (^99^mTc)-MAA and to confirm the coverage of the entire target lesion in the injection area.

During the same session, an angiography was performed through the EHFA to confirm its contribution to the HCC vascularization.

The treatment was carried out within 14 days using ^90^Y, following the same injection pattern as in the workup. The delivered radiation dose was recorded.

When combined with TARE, TACE was performed during the same treatment session.

**Fig. 2:**
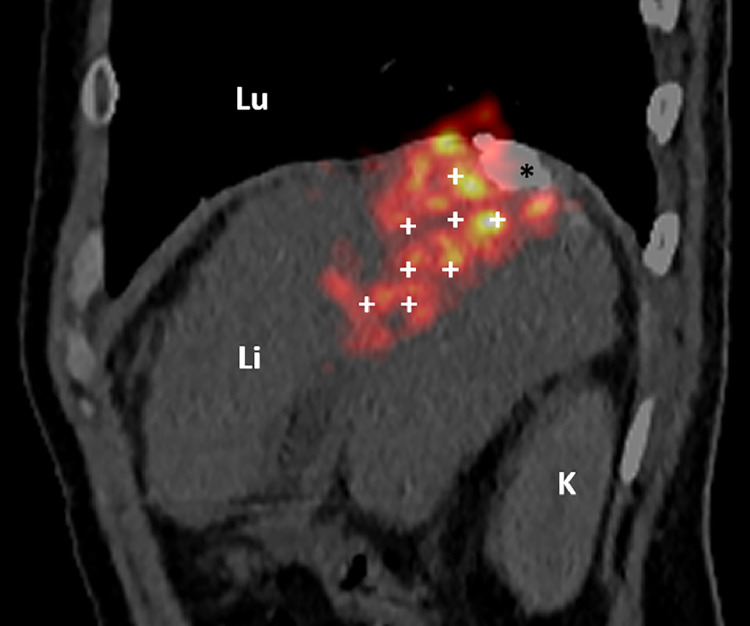
^90^Y PET-CT image showing the treatment with yttrium 90 of the main part of the tumor (white crosses) and the hyperattenuation of the Lipiodol emulsion of the outer part of the lesion (asterisk). Li: liver, Lu: lung, K: kidney.

A ^90^Y PET/CT was performed within 24 h to assess the distribution of yttrium 90 [Fig. 2].

### 2.2 TACE

**Fig. 3:**
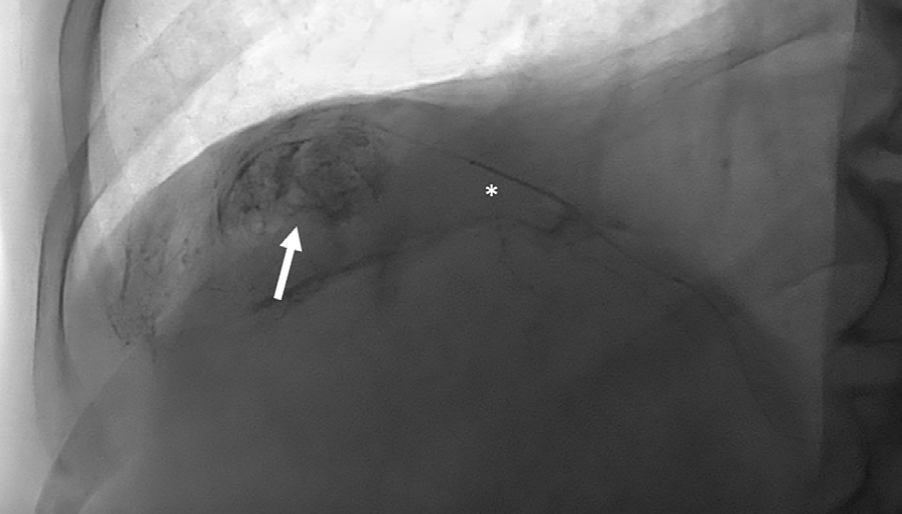
Fluoscopy showing the injection of Lipiodol/doxorubicin emulsion through the microcatheter (asterisk) in the diaphragmatic right artery, vascularising the outer part of the liver lesion (arrow).

**Fig. 4:**
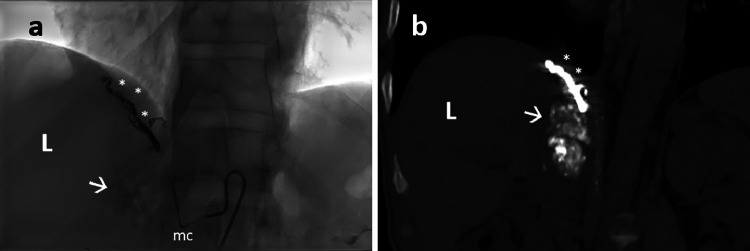
TACE performed through a diaphragmatic right artery. The distal part of the artery was coiled (asterisks) to avoid non target chemoembolization. Then, the emulsion of Lipiodol and doxorubicine was injected (arrow) until flow stasis. a: fluoroscopy image; b: coronal unenhanced CT image after treatment; L: liver; mc: microcatheter.

TACE was executed after catheterization of the EHFA [Fig. 3]. A DP-CBCT was conducted before injection to identify collateral branches not contributing to tumor vascularization. If any were found, they were excluded by coil embolization. The treatment consisted of an emulsion of Lipiodol (10 ml) (Guerbet, France) and doxorubicin (60 mg/9 ml) (1:1) injected until flow stasis or the emulsion was exhausted [Fig. 4]. The embolization was completed with the injection of Embozene 400 μm (Varian Medical Systems, Palo Alto, CA). A non-enhancement CBCT was performed at the end of the procedure to confirm the correct impregnation of the part of the lesion vascularized by the EHFA. All procedures were performed with Allura Clarity and Azurion (Philips, Best, Netherlands).

The volume and main diameter of the part of the lesion treated with TACE were calculated using hyperdensity due to Lipiodol captation on the CBCT obtained at the end of the procedure, and compared to the volume and main diameter of the whole lesion, respectively.

The feasibility of the procedure, defined as the ability to perform a complete TARE and a complete TACE on the same target lesion, was evaluated.

Complications of the procedures were recorded and classified using the SIR scale for adverse events [19]. Safety, defined as the possibility of carrying out the procedure without major complications (i.e., class C-F of the SIR Classification), was also evaluated.

### 2.3 Follow-up

Follow-up was conducted with clinical and imaging evaluations (i.e., MRI or CT scan) one month after the treatment and then every three months. The median duration of follow-up was recorded.

Objective response (OR) of the target lesion was evaluated at 6 months, using mRECIST criteria [20]. Time-to-progression was recorded, as well as any further local or systemic treatment performed after TARE.

### 2.4 Statistics

Due to the non-Gaussian distribution, descriptive statistics were used as appropriate, including proportions and medians with quartiles (first and third quartile).

## 3 Results

Between March 2016 and March 2021, 13 patients with HCC underwent TARE associated with TACE. Four patients were excluded because TACE was performed more than three months after TARE.

Individual tumor dimensions, the presence of portal vein invasion, as well as main clinical and laboratory status at baseline are reported in Table 1. Of the nine remaining patients, there were eight men and one woman, with a median age of 62 years (IQR: 54–72 years). HCC was diagnosed by biopsy in two cases and based on imaging characteristics in seven cases. Cirrhosis was present in seven cases, developing from alcohol abuse in five cases, hepatitis B virus (HBV) in two cases, and hepatitis C virus (HCV) in two cases. Alcohol abuse was associated with HCV in one case and with hemochromatosis in another case. At the clinical evaluation before TARE, six patients were classified as BCLC stage A and three patients as BCLC stage C.

**Table 1:** Morphological characteristics of lesions and clinical and biological characteristics of patients at baseline.

	Liver disease	Tumor volume (cm3)	Lipiodol volume in the tumor (cm3)	Portal vein invasion	Previous treatment on the same nodule	AFP	Clinical status		Laboratory			
							BCLC	Child Pugh	ECOG	Albumine	PT	Bilirubin
***Patient 1***	HCV	213	60	VP 3	TACE	21N	C	A5	0	N	N	N
***Patient 2***	OH	260	75	VP 3	TARE	8N	C	A5	1	N	N	N
***Patient 3***	OH	82	11	VP 0	TARE	N	A	A5	0	N	N	N
***Patient 4***	HCV	75	40	VP 0	TARE	64N	A	A5	0	N	N	N
***Patient 5***	HBV	11	5	VP 0	TARE	485N	A	A5	0	N	N	N
***Patient 6***	HBV	200	24	VP 0		N	A	A5	0	N	N	N
***Patient 7***	OH/Hemocromatosis	300	75	VP 3	TARE	31N	C	A5	0	N	N	N
***Patient 8***	OH	2500	110	VP 0	TACE	N	A	A5	0	N	N	N
***Patient 9***	OH/HCV	67	20	VP 0		11N	A	B8	0	5 < *N*	5 < *N*	2N

HCV: hepatitis C virus; OH: alcohol abuse; HBV: hepatits B virus; TACE: transarterial chemoembolization; TARE: transarterial radioembolization; N: normal value, or multiples of normal value (for example: 8 N, means 8 times the normal values) or units below the normal value (for example: PT 5 < *N* means 65 %). AFP: alpha-fetoprotein, *N* < 10 IU/mL; Albumin: N 32–52 g/l; PT: prothrombin time: N 70–100 %; bilirubin (total): *N* < 20 mmol/l.

Eight patients underwent previous treatments: five patients had undergone TARE and two patients had undergone TACE on the lesion that was later treated with a combination of TACE and TARE, while one patient had a previous contralateral lobectomy. The interval between the treatment of interest and the previous endovascular treatments was at least six months. This interval was justified by the presence of residual active disease observed on post-treatment imaging checks.

The median longest axis of the lesions before treatment was 70 mm (IQR: 60–79 mm), and the median volume of the lesions at that same time was 200 cm³ (IQR: 75–260 cm³). All patients had a single lesion; one patient presented with a satellite nodule of 2 cm that was included in the TARE territory. The lesions were nodular in seven cases and infiltrative in two cases, as determined by their appearance on pre-treatment imaging.

**Fig. 5:**
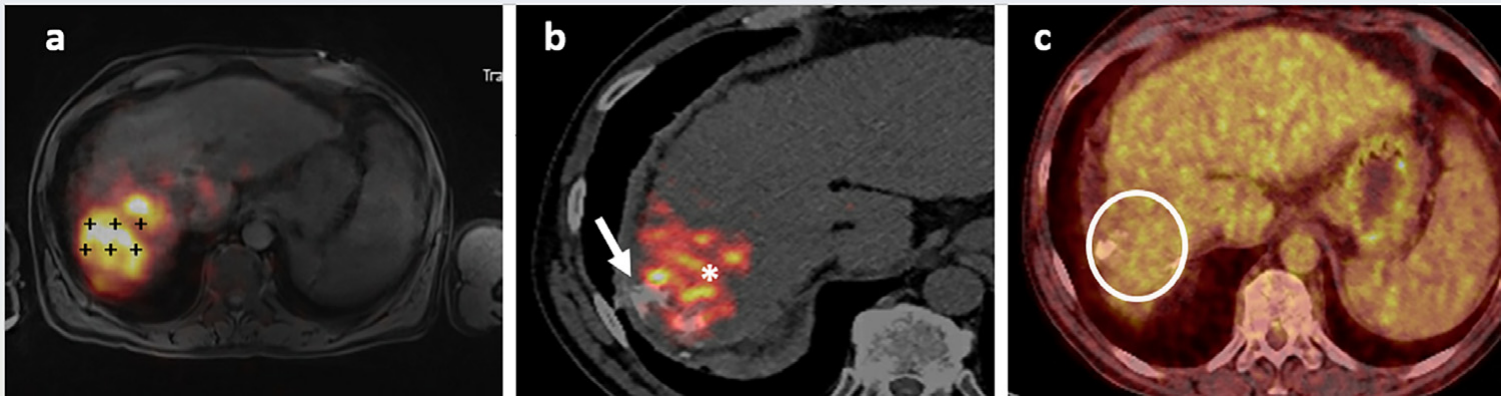
A: PET-MRI axial image showing a HCC of the hepatic dome (black crosses). B: ^90^Y PET-CT image after TARE and TACE showing the accumulation of ^90^Y in the inner part of the lesion (white asterisk) and the Lipiodol hyperattenuation of the outer part of the tumor, treated by TACE through the diaphragmatic artery vessel (white arrow). C: (^18^F)-FDG PET-CT performed 14 months after treatment, showing no residual tumor in the treated area (white circle).

Technical feasibility, defined as the ability to perform TARE through intrahepatic arteries followed by TACE through the EHFA of the same lesion, was achieved in 100 % of the patients (Fig. 5). Six patients underwent TACE and TARE during the same session, while three patients had TACE after TARE: in two cases, it was performed 6 and 13 days after TARE to shorten the duration of the procedure for the patient's comfort. In the third case, TACE was performed 77 days after TARE because the EHFA was not identified on pre-treatment imaging but was suspected after a re-examination of the Y90 PET-CT. In all cases, TARE and TACE targeted the same lesion.

TARE was performed using resin (^90^Y)-labeled microspheres for six patients and glass microspheres for three patients, depending on product availability. The median delivered activity was 935 MBq (IQR: 642–1262 MBq).

The EHFA originated as a collateral branch of the right diaphragmatic artery in six cases, of the right adrenal artery in two cases, and of the left gastric artery in one case. In three cases, coil embolization was performed above the EHFA to avoid non-target TACE (i.e., left intercostal, left adrenal, and left gastric arteries). In all cases, the part of the lesion treated with TACE through the EHFA showed complete Lipiodol retention on the post-injection CBCT.

The volume of the part treated with TACE was 40 cm³ (IQR: 20–75 cm³), and the longest axis was 47 mm (IQR: 35–64 mm). The ratio between the volume of the entire lesion and the part treated with TACE was 3.55 (IQR: 3.35–7.45), while the ratio between the longest axis of the entire lesion and the longest axis of the part treated with TACE was 1.44 (IQR: 1.27–1.7).

Two patients experienced minor complications (grade B), suffering from mild pain, which was managed with simple medical treatment.

In one case, a major complication of grade D occurred, resulting in severe pain and acute on chronic liver failure, requiring intensive care for two days after TACE. Notably, this occurred in the patient with the largest HCC in our series (longest axis: 167 mm) who received the highest radiation dose during TARE (3300 MBq). The TACE, performed through the right diaphragmatic artery, showed a Lipiodol uptake with a long axis of 68 mm. The rest of the population did not experience any complications after treatment. Thus, the procedure was safe in 8 out of 9 cases.

The median follow-up period was 23 months (IQR: 16–29 months). One patient received systemic therapy for extrahepatic progression and radiotherapy for the nodule that had been previously treated with TARE/TACE. This was due to the persistence of a portion of the lesion that remained viable six months after treatment, coupled with anatomical constraints that did not allow for repeat endovascular treatment. Two patients underwent percutaneous thermoablations for newly appeared nodules, and three patients received systemic therapy after developing extrahepatic metastases. In one patient, surgical resection was performed 9 months after TARE treatment, and histological analysis of the surgical specimen revealed no signs of residual disease.

At the 6-month follow-up, concerning the treated lesion, four patients showed complete response (CR), three partial response (PR), and one progressive disease (PD), while one patient was lost to follow-up 1 month after treatment. Concerning the overall disease at the 6-month follow-up, four patients showed CR, three patients showed PD, and one patient had PR [Table 2].

**Table 2:** Tumour response after procedure, including delivered dose during TARE and additional treatments.

	TARE: delivered dose (MBq)	Treatments after TARE	TTP (months)	6-months follow-up	
				Local response	Overall response
*Patient 1*	200*	lobectomy	18	CR	CR
*Patient 2*	1000	Sorafenib	1	PD	PD
*Patient 3*	740	PA of new nodules	13	CR	CR
*Patient 4*	870	PA of new nodules	22	CR	CR
*Patient 5*	350	A-B	4	PR	PD
*Patient 6*	ND*	no other treatment	no progression	PR	PR
*Patient 7*	1900	A-A	13	CR	CR
*Patient 8*	3300	A-B	2	PR	PD
*Patient 9*	1050*	no other treatment	ND	ND	ND

*: treatment performed with glass microspheres; PA: percutaneous ablation; TTP: time-to-progression; A-B: atezolizumab–bevacizumab; A-A: atezolizumab–avastin; CR: complete response; PD: progression disease; PR: partial response; ND: not defined.

Regarding the overall disease, the median time to progression was 13 months (IQR: 3.5–19 months).

## 4 Discussion

The main finding of this article is that nodules of hepatocellular carcinoma HCC fed by both intra- and extra-arterial supplies can be treated with a combination of intrahepatic transarterial radioembolization TARE and TACE. In our series of nine patients, the combined treatment was feasible in all cases. Extrahepatic TACE was carried out through the right diaphragmatic artery in six cases, the right adrenal artery in two cases, and the left gastric artery in one case. An objective local response was obtained in seven cases at the 6-month follow-up, even though four patients underwent systemic treatment after the local treatment due to the development of distant metastases.

Several studies have demonstrated the effectiveness of TACE performed through extrahepatic arteries [21], [22], [23], with variable results in terms of tumor response and clinical safety. Similarly, other authors have reported their experiences and techniques for redistributing arterial flow to lesions with EHFAs but aimed to be treated with TARE [12,14].

Ezponda et al. [14] reported a 40 % rate of suboptimal flow redistribution after EHFAs coiling, possibly due to the opening of other shunts after simple coil embolization, contributing to dispersed flow. Moreover, in cases of persistent hypervascularization, no additional transarterial options are available through the extrahepatic feeding branches once the coils are delivered.

Abdelmaksoud et al. [12] reported a 93 % success rate in redirecting arterial flow to the intrahepatic vessels after embolization with particles of the EHFA. The use of microparticles likely allows for more distal embolization, preventing the opening of new shunts and enabling a more effective redistribution of the intrahepatic flow. Additionally, a certain embolizing power can be hypothesized, even though the authors consider that the particles were too large to induce necrosis. More likely, the particles were responsible for a bland embolization of the external border of the lesion. Our technique is somewhat an evolution of this: the tumor burden vascularized by the EHFA is relatively small, with a median diameter of 47 mm, which is not different from the diameter of a lesion typically treated with TACE in an ordinary setting [5,11,24].

Furthermore, extrahepatic TACE could have the dual role of treating the border of the tumor and inducing the redistribution of flow. This hypothesis cannot be confirmed by our results due to the small sample size. Therefore, more studies are needed to confirm this hypothesis. On the other hand, TACE is not only a way to redistribute the flow but also a treatment in itself, which can be proposed even as a second step, in case of late evidence of incomplete distribution of ^90^Y over the entire lesion [25,26], as was the case in two patients in our series.

Burgmans et al. [27] described the performance of TARE through EHFAs, but out of 25 patients, TARE was feasible in only five cases. The reasons for not performing TARE in the remaining cases were mainly related to the small tumor supply provided by the extrahepatic artery, shunt/non-target enhancement, and failed catheterization. Thus, this option remains rarely accessible.

A major complication was found in one case, and it was related to the size of the treated lesion. Therefore, even though our series is too small to establish a conclusive cutoff, we can assume that the limitation of the treatment is related to the dose delivered to the lesion and to the volume of the lesion itself, as is the case for intrahepatic treatment.

Our study has several limitations: first, the small number of patients and the monocentric retrospective nature of the study. No solid statistical conclusions can be drawn due to the low number of cases and the heterogeneity of the patients, considering both the type of lesion and the underlying liver disease. Additionally, the procedural approach is not homogeneous, as some patients had TACE after TARE. Therefore, results can be influenced by the sequence of treatment. Lastly, TARE was performed with both glass and resin beads, which can have different patterns of distribution, especially in cases of large lesions.

## 5 Conclusions

For HCC lesions with both intrahepatic and extrahepatic artery supply, the combination of transarterial chemoembolization TACE through extrahepatic feeding arteries, in addition to transarterial radioembolization TARE performed through intrahepatic arteries, is technically feasible. Preliminary data show promising safety and local tumor control. Further studies are warranted to validate the promising results of this new therapeutic option for treating HCC in cases where EHFAs supply the lesion.

## Author role statement

LC Pescatori and A Galletto conceived the study and collected the data. LC Pescatori wrote the article. V Tacher supervised and validate the drafting. Other authors reviewed the article, checked the accuracy and edited the draft.

## Ethical statements

*Ethical approval and consent to participate:* Informed consent was obtained from every patient and medical ethics approval was waived, due to the retrospective nature of the study.

*Consent for publication:* For this type of study consent for publication is not required.

*Availability of data and materials:* on request.

## Funding

This research did not receive any specific grant from funding agencies in the public, commercial, or not-for-profit sectors.

## Declaration of competing interest

Authors declare that they do not have any conflict of interest.
